# Eupalinolide B inhibits hepatic carcinoma by inducing ferroptosis and ROS-ER-JNK pathway: Inhibition of Eupalinolide B in hepatic carcinoma

**DOI:** 10.3724/abbs.2022082

**Published:** 2022-07-18

**Authors:** Yonghui Zhang, Haoyang Zhang, Jinage Mu, Meiyue Han, Zhihao Cao, Feng Dong, Tingting Wang, Lian Pan, Wujing Luo, Jiaxin Li, Huan Liu, Lishan Jin, Wenxuan Ding, Yong Wei, Xuesong Deng, Dan Liu, Xiuzhen He, Yi Pang, Xiao Mu, Zhongjun Wu, Dilong Chen

**Affiliations:** 1 The First Affiliated Hospital of Chongqing Medical University Chongqing 400042 China; 2 The People’s Hospital Affiliated to Chongqing Three Gorges Medical College Chongqing 404100 China; 3 Chongqing Key Laboratory of Development and Utilization of Genuine Medicinal Materials in Three Gorges Reservoir Area Chongqing 404120 China; 4 Chongqing Engineering Research Center of Antitumor Natural Drugs Chongqing 404120 China; 5 Key Laboratory of Intelligent Information Processing and Control College of Electronic and Information Engineering Chongqing Three Gorges University Chongqing 404110 China

**Keywords:** Eupalinolide B, hepatic carcinoma, ferroptosis, HO-1, ER stress, JNK

## Abstract

Primary hepatic carcinoma is a common malignant tumor. The classic molecular targeted drug sorafenib is costly and is only effective for some patients. Therefore, it is of great clinical significance to search for new molecular targeted drugs. Eupalinolide B (EB) from *Eupatorium lindleyanum* DC. is used to treat chronic tracheitis in clinical practice. However, the role of EB in hepatic carcinoma is unknown. In this study, we first measure the effect of EB on tumor growth in a xenograft model and PDX model. The cell proliferation and migration are also detected in human hepatocarcinoma cell lines (SMMC-7721 and HCCLM3). Then, we investigate cell cycle, cell apoptosis, cell necrosis, cell autophagy, and ferroptosis by flow cytometry, western blot analysis and electron microscopy. The results demonstrate that EB exerts anti-proliferative activity in hepatic carcinoma by blocking cell cycle arrest at S phase and inducing ferroptosis mediated by endoplasmic reticulum (ER) stress, as well as HO-1 activation. When HO-1 is inhibited, EB-induced cell death and ER protein expression are rescued. The migration-related mechanism consists of activation of the ROS-ER-JNK signaling pathway and is not connected to ferroptosis. In summary, we first discover that EB inhibits cell proliferation and migration in hepatic carcinoma, and thus EB is a promising anti-tumor compound that can be used for hepatic carcinoma.

## Introduction

Primary hepatic carcinoma is one of the most common malignant tumors in the world, and according to 2021 Cancer Statistics, the incidence of hepatic carcinoma continues to rise. Because the five-year survival rate of 20% for hepatic carcinoma is among the lowest for cancers [ 1, 2] , there is an urgent need to explore effective treatments for hepatic carcinoma. The toxicity and side effects of radiotherapy and chemotherapy are obvious, and the life quality of patients is very poor [3]. Molecular targeted therapy plays an important role in treating hepatic carcinoma, although currently sorafenib is the only agent approved by the US FDA for treating primary hepatic carcinoma. The use of sorafenib is accompanied by some disadvantages such as only effective for some patients, severe adverse effects, and high cost [ 4– 6] . Therefore, the search is ongoing for new molecular targeted drugs, and an increasing number of scientists are beginning to explore natural products as molecular targeted drugs to treat liver cancer.

The aerial parts of Eupatorium ( *Eupatorium lindleyanum* DC.; also called Ye-Ma-Zhui) are used to resolve phlegm and relieve cough, clear heat and detoxify, reduce blood pressure to treat chronic tracheitis, bronchitis, and hypertension [ 7– 11] . Studies have shown that Eupatorium extract exerts anti-cancer effects against breast and lung cancers [ 12, 13] . Eupalinolide B (EB) is a sesquiterpene lactone constituent extracted from Eupatorium [14], and there is no study on the effect of EB on hepatic carcinoma.

In this study, we investigated the pharmacological activities of EB on tumor growth in human hepatic carcinoma xenograft model and hepatocarcinoma cell proliferation *in vivo* and *in vitro*, and investigated the mechanisms including the cell cycle, cell apoptosis, cell necrosis, cell autophagy, and ferroptosis. The results suggest that EB plays a role in inhibiting hepatic carcinoma by inducing ferroptosis and blocking the cell cycle of hepatic cancer cells.

## Materials and Methods

### Reagents and antibodies

Roswell Park Memorial Institute (RPMI)-1640 medium, Dulbecco’s modified Eagle’s medium (DMEM) and fetal bovine serum (FBS) were purchased from Gibco (New York, USA). Penicillin and streptomycin (P/S) was purchased from Invitrogen (Carlsbad, USA). EB (A0660; molecular formula: C24H30O9; relative molecular mass: 462.29) was purchased from Must Bio-technology (Chengdu, China). Z-VAD-FMK (apoptosis inhibitor), necrostatin-1 (necroptosis inhibitor), autophagy inhibitor 3-MA (autophagy inhibitor), zinc protoporphyrin (ZnPP, HO-1 inhibitor), 4-phenylbutyric acid (4-PBA, ER stress inhibitor), SP600125 (JNK pathway inhibitor), SB203580 (p38 pathway inhibitor) and U0126 (MEK 1/2 inhibitor) were purchased from MedChemExpress LLC (New Jersey, USA). N-acetyl-L-cysteine NAC (ROS scavenger), ferrostatin-1 (Ferr-1, ferroptosis inhibitor), deferoxamine (DFO, iron chelator), agarose and BrdU were purchased from Sigma-Aldrich (St Louis, USA). Goat serum was purchased from ZSGB-Bio (Beijing, China). CCK-8 solution was purchased from Dojindo Molecular Technologies (Shanghai, China). Alexa FluorR® 594 goat anti-rat IgG secondary antibody was purchased from Life Technologies (New York, USA). Polycarbonate membrane inserts (8-μm pore size) was purchased from Corning Co. (Corning, USA). Nude mice (BALB/c) were purchased from the Beijing Laboratory Animal Research Center (Beijing, China). Cell cycle detection kit was purchased from KeyGEN BioTECH (Nanjing, China). Cell apoptosis detection kit was purchased from NeoBioscience (Nanjing, China). TRNzol Universal RNA Reagent was purchased from Tiangen Biotech (Beijing, China). BODIPY™ 581/591 C11 was purchased from Thermo Fisher Scientific (Waltham, USA). Radioimmunoprecipitation (RIPA) lysis buffer, horseradish peroxidase (HRP)-conjugated goat anti-mouse IgG and goat anti-rabbit IgG secondary antibodies were purchased from Beyotime (Shanghai, China). Polyvinylidene difluoride (PVDF) membranes was purchased from Millipore (Burlington, USA). CDK2 (2546T), cyclin E1 (20808S), E-cadherin (14472), vimentin (5741T), cleaved PARP (5625T), cleaved caspase-3 (9611T), CHOP (2895), Bip (3177), MAPK family sampler kit (9926T), p-ERK (4370T), p-JNK (4668T), p-p38 (4511T) and LC3B (12741) were purchased from Cell Signaling Technology (Danvers, USA). GRP78 (sc376768), IRE1a (sc390960) and ATF-6α (sc166659) were purchased from Santa Cruz Biotechnology. Anti-BrdU primary antibody, GPX4 (ab125066) and HO-1 (ab13248) were purchased from Abcam (Cambridge, England). RIP1 (CY6582), p-MLKL (CY7146) and MLKL (CY5493) were purchased from Abways (Shanghai, China). β-actin (TA-09) was purchsed from Zhongshan Goldenbridge Bio (Beijing, China). SYBR Green Master Mix:SYBR®Premix Ex Taq™II (Tli RNaseH Plus) was purchased from TaKaRa (Tokyo, Japan).

### Cell culture

Human hepatic carcinoma cell lines SMMC-7721 and HCCLM3 were purchased from Zhong Qiao Xin Zhou Biotechnology (Shanghai, China). SMMC-7721 cells were cultured in RPMI-1640 medium, and HCCLM3 cells were cultured in DMEM. The media were supplemented with 10% fetal bovine serum together with 1% penicillin and streptomycin (P/S). The cells were cultured under standard conditions of 5% CO _2_ at 37°C.

### Eupalinolide B treatment

EB was dissolved in dimethyl sulfoxide (DMSO) to make a 40 mM stock solution. EB at different concentrations (6, 12, and 24 μM) was then used to treat the hepatic carcinoma cell lines SMMC-7721 and HCCLM3, with DMSO being used as a control. Exposure to EB was performed for different time periods (0, 6, 24, 48, and 72 h). All of the experiments were independently performed in triplicate.

### Cell morphology observation

Cells were visualized and photographed using a phase-contrast microscope equipped with a digital camera (Leica Microsystems, Wetzlar, Germany).

### Cell viability assay

Cell counting kit-8 (CCK-8) assay was performed to measure cell viability. SMMC-7721 and HCCLM3 cells were cultured to a confluence of approximately 90%, and the cells were then counted. The cells were seeded into 96-well culture plates at 5×10 ^3^ cells/well. EB with a final concentration of 6, 12, or 24 μM was added to the drug administration groups, the cells were cultured for 24, 48, or 72 h, and then, 10 μL CCK-8 solution was added. The cells were incubated in a 37°C incubator for 4 h, and the OD value was then measured at 450 nm using a microplate spectrophotometer (ELX800 ^TM^; BioTek, Winooski, USA) for data analysis.

### Bromodeoxyuridine (BrdU) staining

Cell proliferation was monitored by BrdU staining. First, 2×10 ^4^ cells in logarithmic phase were seeded in 24-well plates and then attached overnight in a 37°C incubator. The cells were then treated with medium containing 24 μM EB, and DMSO was used as a control. After 48 h, the cells were treated for 2 h with 10 μg/mL BrdU, and then, the cells were fixed with 4% paraformaldehyde for 15 min. After treatment with 2 M HCl and then with 0.3% Triton X-100, cells were blocked with 10% goat serum. Cells were then successively incubated with anti-BrdU primary antibody and then with Alexa FluorR® 594 goat anti-rat IgG secondary antibody. The cell nuclei were stained with DAPI, and BrdU-positive cells in random fields were counted under a fluorescence microscope (DMI8; Leica, Wetzlar, Germany).

### Soft agar assay

The soft agar assay was used to measure the colony formation ability of hepatic carcinoma cells. Six-well plates were filled with base agar consisting of 1.5 mL of 2×RPMI-1640 or 2×DMEM in 0.6% agarose. One thousand cells in logarithmic phase were mixed with medium containing 1.2% agar as well as 24 μM EB, and were subsequently added as an additional layer on top of the base agar. After being cultured in a 37°C incubator for 21 days, images of colonies were captured under a microscope and the number of colonies was counted after staining with MTT [15].

### Tumor xenografts

Four-week-old nude mice (BALB/c) were used, and they were housed for 1 week in a specific pathogen-free (SPF) environment before experimentation. Each mouse received subcutaneous injections of 1×10 ^6^ SMMC-7721 or HCCLM3 cells in 200 μL phosphate-buffered saline (PBS) into both flanks. One group was intraperitoneally injected every two days with EB at 25 or 50 mg/mouse body weight, for a total of 3 weeks. As a control, DMSO was used in another group. Tumor size was measured to calculate tumor volume, and mouse body weights were monitored every two days. Three weeks after injection of cells, the mice were euthanized, and tumors were excised and weighed [16].

### Patient-derived tumor xenograft (PDX) model

Tissue samples removed from patients with liver cancer were placed into UW preservation solution immediately after they were isolated. Tumors were cut into 2 ×2×2 mm pieces and then subcutaneously inoculated into 4-week-old male BALB/c nude mice. One group was intraperitoneally injected every two days with EB at 50 mg/mouse body weight, for a total of 3 weeks. As a control, DMSO was used in another group. The mice were euthanized, and tumors were excised and weighed. The obtained tumor tissues were further subject to western blot analysis [17].

### Transwell assay

The migration assay was conducted in a 24-well Transwell cell culture apparatus fitted with polycarbonate membrane inserts (8-μm pore size). Briefly, 2×10 ^5^ cells were seeded into the upper chamber of the insert with 200 μL of 1% serum, and 500 μL media containing 10% FBS were added into the lower chamber as a chemoattractant. All of the media contained 24 μM EB or DMSO. After incubation at 37°C in a 5% CO _2_ incubator for 24 h, the chambers were rinsed three times with PBS, and nonmigrating cells were removed from the top wells with cotton swabs. Cells on the lower membrane surface were fixed with 4% paraformaldehyde and then stained with 0.5% crystal violet. Migrated cells were treated with 33% acetic acid, and the absorbance was measured at 560 nm. Each experiment was performed in triplicate, and the migration rate was normalized by the proliferation rate.

### Cell scratching assay

Cells were cultured in 24-well plate and reached full of confluence, then the monolayer of the cells were scratched using a yellow pipette tip. Subsequently, PBS was used to wash and remove floating and damaged cells, and serum-free medium with 24 μM EB or DMSO were added to cells for culture. Cells migrated over the denuded area were observed and pictures were taken at indicated times. The corporation of wound closure was measured at the indicated time points.

### Flow cytometric analysis

Cells were cultured in medium containing 12 or 24 μM EB, and DMSO was used as a control. Then cells were harvested for flow cytometric analysis performed according to instructions. For the cell cycle assay, cells were washed with cold phosphate-buffered saline (PBS) and then fixed with 75% ethanol at 4°C for 24 h. After being washed twice with PBS, cells were incubated in 500 μL PBS containing propidium iodide (PI) and RNase A (9:1) at 37°C for 60 min. Cells were then analyzed on a DxFLEX flow cytometer (Beckman Coulter, Brea, USA). For cell apoptosis analysis, an Annexin V-FITC/PI apoptosis assay kit was used. The cells treated with EB were collected at 48 h and washed twice with cold PBS. Cells were then suspended at a density of 5×10 ^5^ cells/mL with 200 μL binding buffer. Next, 195 μL binding buffer and 5 μL Annexin V-FITC were added and incubated for 10 min at room temperature. The cells were washed, suspended in binding buffer, and then stained with PI. All of the experiments were independently performed in triplicate. Flow cytometry as well as Modfit and CytExpert software were used to analyze the cell cycle, apoptosis, and ROS of hepatic carcinoma cells.

### RNA sequencing and data analysis

Human liver cancer cells were treated with EB (24 μM) or DMSO, and samples were collected using TRNzol Universal RNA Reagent and stored at −80°C. Samples were sent to Personalbio Biological Technology (Shanghai, China) for transcriptome sequencing. The samples underwent RNA extraction, purification, and database construction using second-generation sequencing technology, based on the Illumina sequencing platform. The raw data were filtered, and the filtered sequences were compared with the human reference genome. According to the comparison results, the expression level for each gene was calculated, and then, the expression difference, enrichment, and clustering analyses were performed on the samples.

### Oxidative stress measurement

The oxidative stress was assessed by fluorescence assay using the fluorophore BODIPY™ 581/591 C11. Cells were cultured in medium containing 24 μM EB or 5 μM Erastin. Fluorescent probes were added to the treated cells. The cells were incubated for 30 min at 37°C with C11-BODIPY 581/591. Subsequently, the cells were washed with PBS twice to remove the unbound dye. Nuclear morphological features of cells were detected after staining with Hochest33342. Fluorescence images were observed under a DMI8 fluorescence microscope.

### Western blot analysis

Cells were lysed in radioimmunoprecipitation (RIPA) lysis buffer. Cell lysates were denatured by dry thermostatic metal bath (Voshin, Wuxi, China) at 100°C for 5 min. Proteins were separated by 10% or 12% sodium dodecyl sulfate-polyacrylamide gel electrophoresis (SDS-PAGE), and then transferred onto polyvinylidene difluoride (PVDF) membranes. The membranes were then blocked in 5% BSA at room temperature for 2 h. Primary antibodies against CDK2 (1:1000), cyclin E1 (1:1000), E-cadherin (1:1000), vimentin (1:1000), cleaved PARP (1:1000), cleaved caspase-3 (1:1000), CHOP (1:1000), Bip (1:1000), MAPK family sampler kit (1:1000), p-ERK (1:1000), p-JNK (1:1000), p-p38 (1:1000), LC3B (1:1000), GRP78 (1:500), IRE1a (1:500), ATF-6α (1:500), GPX4 (1:1000), HO-1 (1:1000), RIP1 (1:1000), p-MLKL (1:1000), MLKL (1:1000), and β-actin (1:1000) were incubated with membranes at 4°C overnight. Next, membranes were incubated with the corresponding horseradish peroxidase (HRP)-conjugated goat anti-mouse IgG or goat anti-rabbit IgG, secondary antibodies (1:10000) at room temperature for 2 h. Proteins were finally visualized using the enhanced chemiluminescence (ECL) system (Bio-Rad, Hercules, USA), and then images were captured by the ChemiDoc MP Imaging System (Bio-Rad). The integrated optical density value of the protein bands was determined by Bio-Rad Image Lab Software, and β-actin was used as an internal reference [18].

### Quantitative real-time reverse transcription PCR (qRT-PCR)

Total RNA was extracted using TRNzol Universal RNA Reagent. Then, 5 μg of total RNA was used as a template for cDNA strain synthesis using a RevertAid First Strand cDNA Synthesis Kit according to the manufacturer’s instructions. The qRT-PCR analysis was performed on the ABI 7500 Real Time PCR System (Applied Biosystems Inc., Waltham, USA) using SYBR Green Master Mix:SYBR®Premix Ex Taq™II (Tli RNaseH Plus) (TaKaRa, Tokyo, Japan). The expression levels of mRNA were normalized against the level of *β-actin* mRNA in the same sample. The primers are listed as follows: *HO-1* forward primer: 5′-CGCCTTCCTGCTCAACATT-3′ and reverse primer: 5′-TGTGTTCCTCTGTCAGCATCAC-3′; *β-actin* forward primer: 5′-GATCTGGCACCACACCTTCT-3′ and reverse primer: 5′-GGGGTGTTGAAGGTCTCAAA-3′.

### Statistical analysis

GraphPad software was used for statistical analyses. Data are presented as the mean±SD. Significant difference was computed by Student’s *t*-test. *P*<0.05 was considered to be statistically significant.

## Results

### EB inhibits tumor growth and clonogenicity in hepatic carcinoma cells

**Figure 1:**
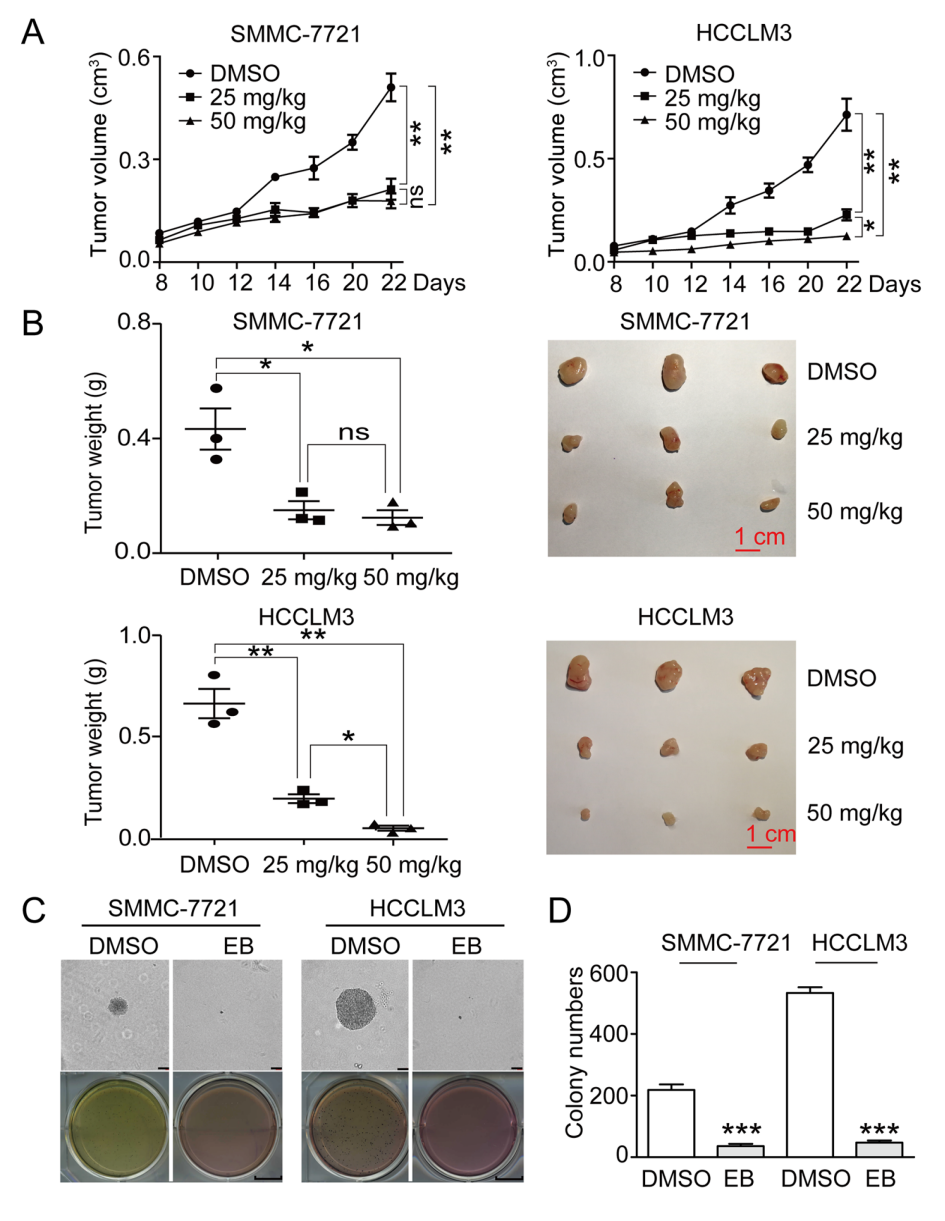
Figure 1 EB suppresses tumor growth in xenograft model of human hepatic carcinoma cells (A) SMMC-7721 and HCCLM3 tumor volume. (B) The weight of xenograft tumors formed by the SMMC-7721 and HCCLM3 cells. (C) Soft agar assays of the colony formation abilities of SMMC-7721 and HCCLM3 cells. Scale bar= 100 μm. (D) Colony numbers in panel (C) were quantified. n=3. All data are shown as the mean±SD. * P<0.05, ** P<0.01, *** P<0.001.

We first established a subcutaneous liver cancer model in nude mice. In this mouse model, SMMC-7721 or HCCLM3 cells were subcutaneously transplanted into female BALB/c nude mice. EB (25 mg/kg or 50 mg/kg) was injected every 2 days for 3 weeks after the tumor expanded. The results showed that EB significantly inhibited tumor volume ( Figure 1A). Figure 1B showed that EB remarkably inhibited the tumor weight in nude mice. The results were verified in PDX model ( Supplementary Figure S1A,B). To assess the effect of EB on cell colony formation, we employed a soft agar assay *in vitro*. The results showed that there were smaller and fewer colonies of EB-treated cells as compared to the control groups ( Figure 1C,D). These data indicated that EB inhibited tumor growth and clonogenicity in hepatic carcinoma cells.

### EB inhibits the proliferation of human hepatic carcinoma cells *in vitro*

**Figure 2:**
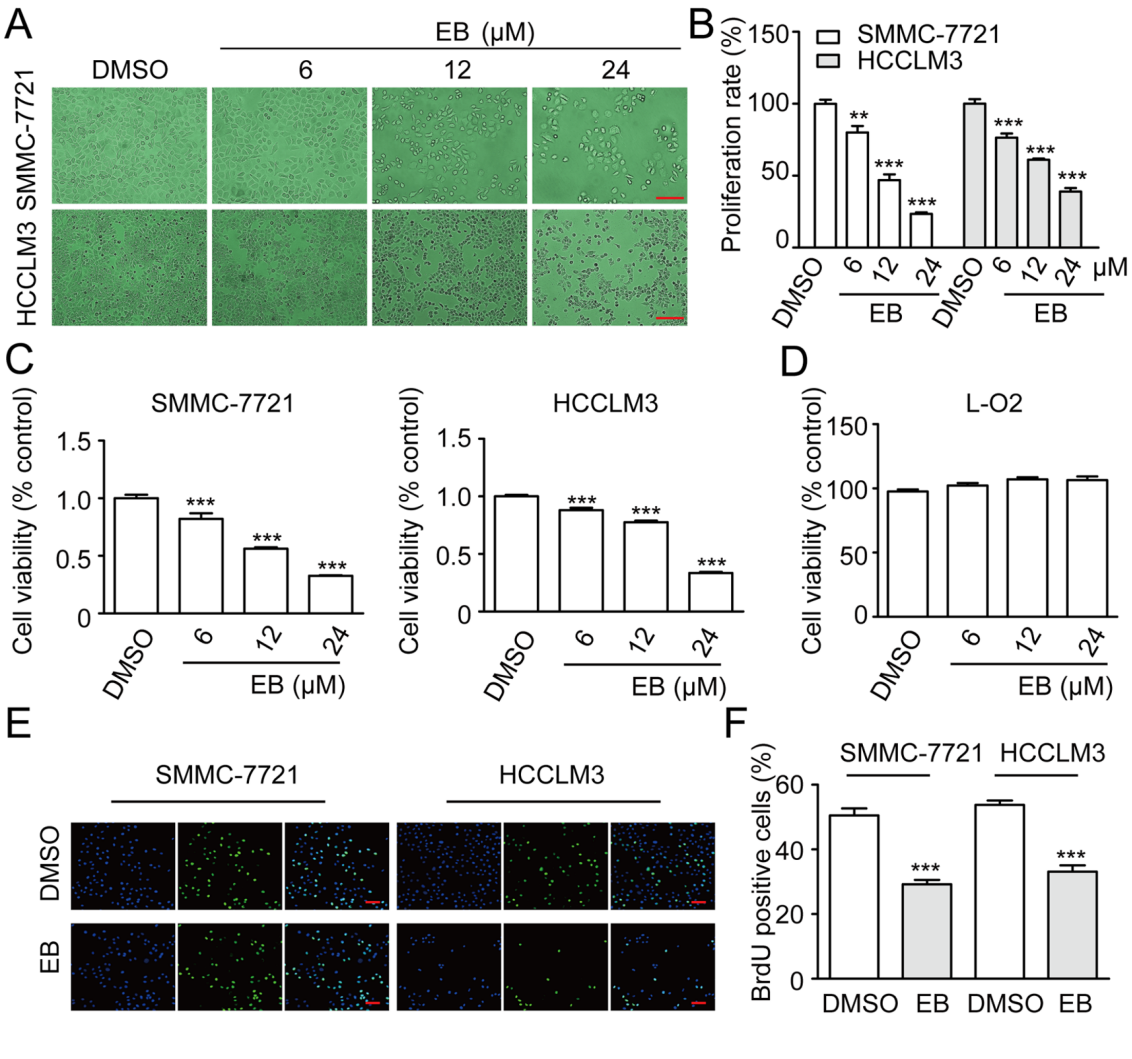
Figure 2 EB inhibits cell growth in human hepatic carcinoma (A) The morphology of SMMC-7721 and HCCLM3 cells. Scale bar =100 μm. (B) Relative numbers of SMMC-7721 and HCCLM3 cells. Cell numbers of DMSO-treated group were regarded as 100%. n=3. (C) Viability of SMMC-7721 and HCCLM3 cells. n=6. (D) The cell viability of L-O2 cell. n=6. (E) Images of SMMC-7721 and HCCLM3 cells with BrdU staining. Scale bar=100 μm. (F) The histogram demonstrates the numbers of BrdU-positive SMMC-7721 and HCCLM3 cells. n=3. All data were presented as the mean±SD. * P<0.05, ** P<0.01, *** P<0.001.

To determine the effect of EB on hepatic carcinoma cells *in vitro*, we treated human hepatic carcinoma cells, SMMC-7721 and HCCLM3, with different concentrations of EB (6 μM, 12 μM, and 24 μM) or DMSO as a control for 48 h. It was found that there were significant morphological changes and decreased numbers of SMMC-7721 and HCCLM3 cells in a dose-dependent manner upon exposure to EB ( Figure 2A,B). The Cell Counting Kit-8 (CCK-8) assay showed sharp declines in the growth of both human hepatic carcinoma cells with treatment of EB (6 μM, 12 μM, and 24 μM) for 48 h, compared with the DMSO group ( Figure 2C). To verify that EB could be appropriately used as a target drug, we measured the effect of EB on the human normal liver cell line L-O2. According to the CCK-8 assay, obvious toxicity was not observed after treatment of L-O2 cells with EB ( Figure 2D). The bromodeoxyuridine (BrdU) assay showed that DNA synthesis was decreased in the group treated with 24 μM EB for 48 h ( Figure 2E,F). These results suggested that EB selectively inhibited human hepatic carcinoma cell proliferation without affecting the normal liver cell line.

### EB inhibits cell migration in human hepatic carcinoma cells

**Figure 3:**
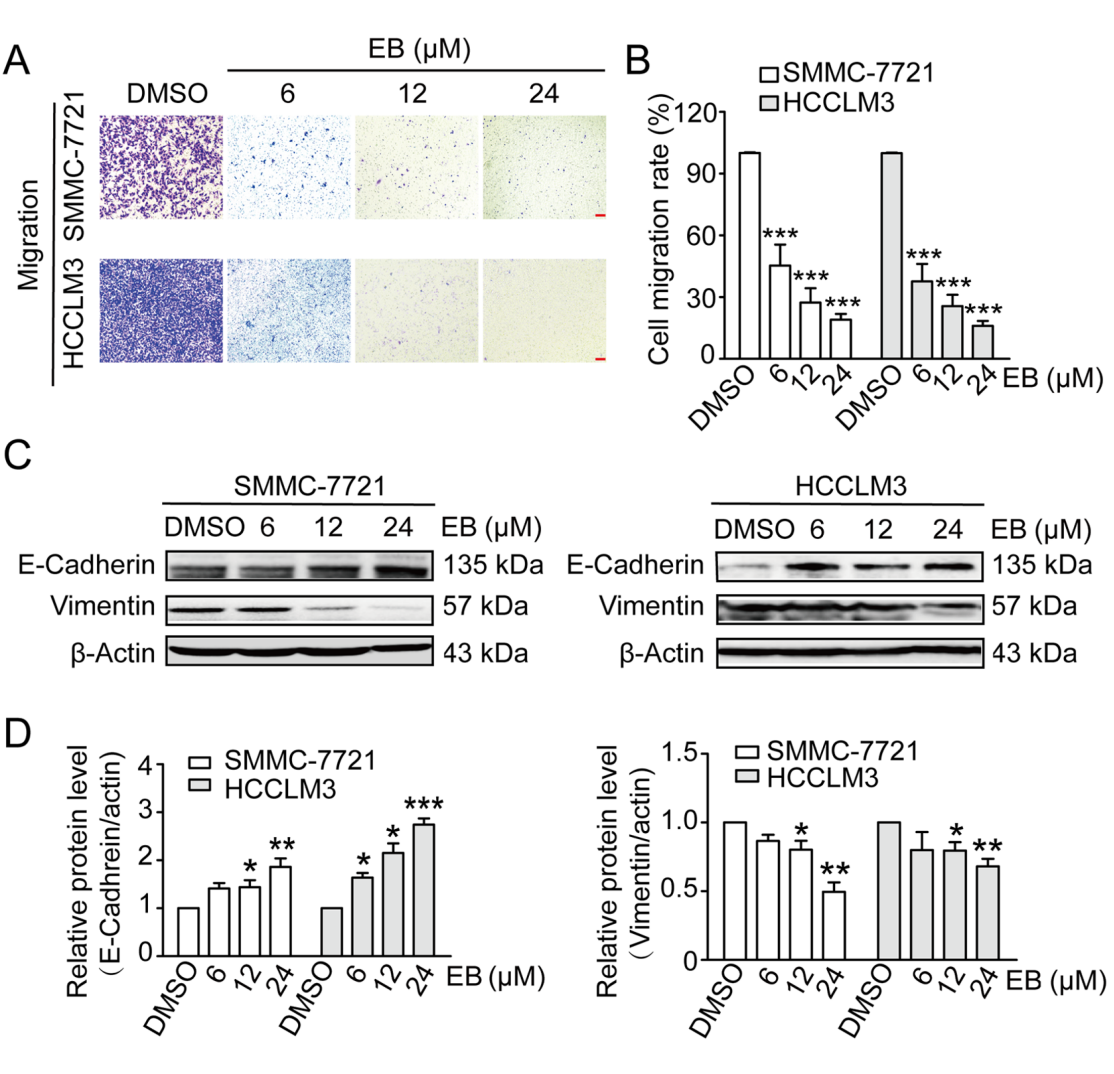
Figure 3 EB inhibits cell migration in human hepatic carcinoma (A) Transwell migration assays of SMMC-7721 and HCCLM3 cells treated with DMSO, 12 or 24 μM EB for 24 h. n=3. (B) The statistical analysis is presented in histograms, and the migration rates were normalized by the proliferation rate. (C) Western blot analysis of the EMT-related protein levels at 48 h in SMMC-7721 and HCCLM3 cells respectively. Cells were treated with the indicated concentration of EB for the indicated time; β-Actin was used as a control. (D) The statistical analysis is presented. n=3. All data are shown as the mean±SD. * P<0.05, ** P<0.01, *** P<0.001.

The effects of EB on the migration abilities of human hepatic carcinoma cells were also evaluated via transwell migration assay. SMMC-7721 and HCCLM3 cells were used in these assays, and similar results were obtained. Here, data from SMMC-7721 and HCCLM3 cells are shown. The results of transwell assay showed that the migration rate of SMMC-7721 cells was decreased by 38.29%±0.49% and 38.48%±0.84% after treatment with EB (12 μM and 24 μM, respectively). Similarly, the migration rate of HCCLM3 cells was decreased by 43.83%±1.08% and 53.22%±0.36% after treatment with EB (12 μM and 24 μM, respectively) ( Figure 3A,B).

The expression levels of E-cadherin and vimentin were evaluated by western blot analysis. The results showed that E-cadherin expression was significantly upregulated after treatment with EB in a dose- and time-dependent manner. However, vimentin expression was remarkably downregulated ( Figure 3C,D). These results suggested that EB treatment reversed the epithelial-mesenchymal transition (EMT) of human hepatic carcinoma cells. Therefore, our results indicate that EB can strongly inhibit the cell migration abilities of human hepatic carcinoma cells.

### EB causes cell cycle arrest at S phase

**Figure 4:**
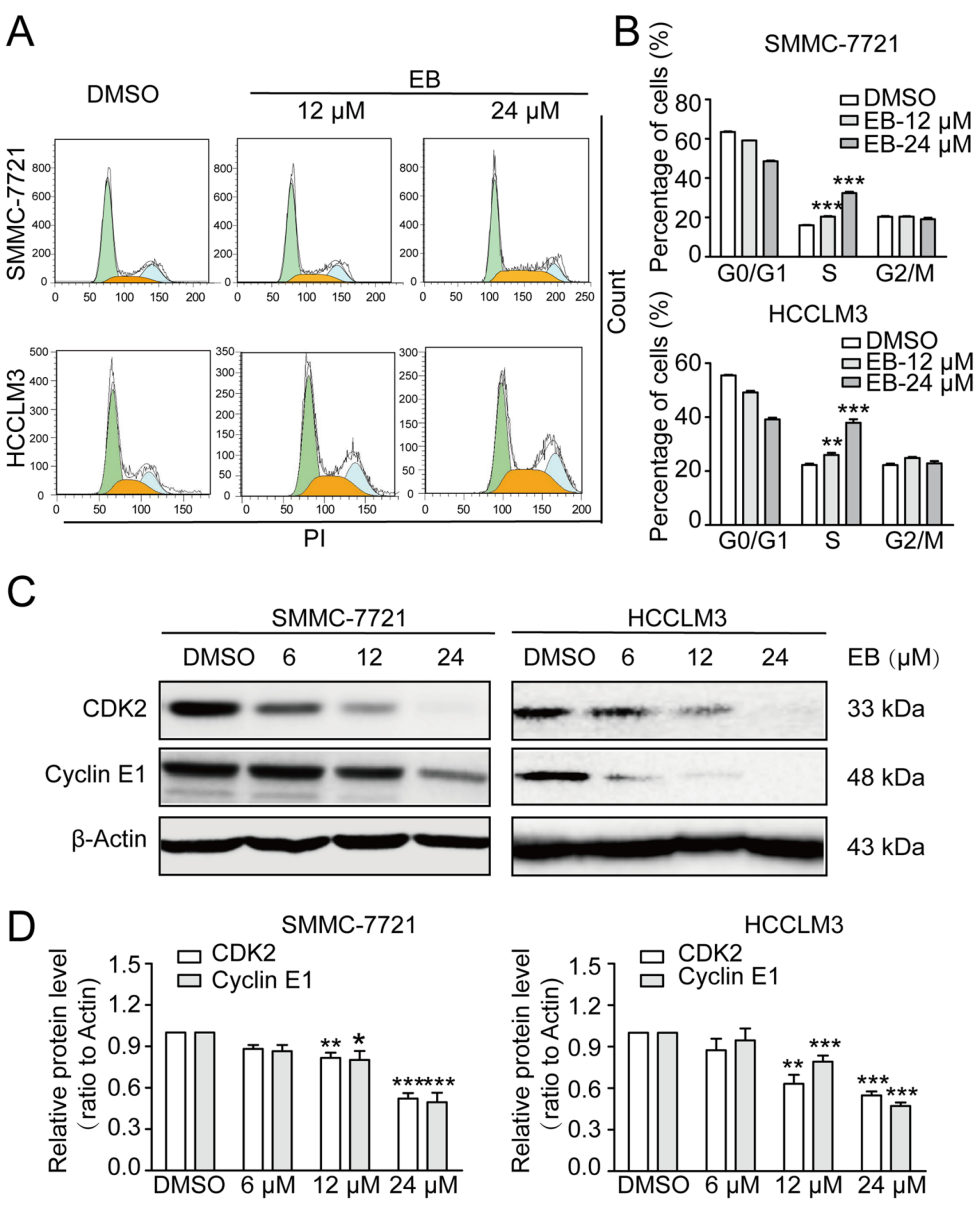
Figure 4 EB induces cell cycle arrest at the S phase (A) Cell cycle of SMMC-7721 and HCCLM3 cells analyzed by flow cytometry. (B) Percentage of SMMC-7721 and HCCLM3 cells in different phases. n=6. (C) Representative western blots of cell cycle-related proteins, CDK2 and Cyclin E1. (D) Densitometry of western blots in panel (C). n=3. All data are shown as the mean±SD. * P<0.05, ** P<0.01, *** P<0.001.

We further analyzed the cell cycle by flow cytometry to evaluate whether EB inhibits cell proliferation by blocking the cell cycle. The results showed that the percentages of S phase in SMMC-7721 and HCCLM3 cells were significantly increased following treatment with 12 μM and 24 μM EB for 48 h ( Figure 4A,B), which indicates that EB causes cell cycle arrest at the S phase. To confirm these findings, we examined CDK2 and cyclin E1 proteins that are essential for G1/S phase transition. The treatment with EB at 12 μM and 24 μM for 48 h significantly decreased CDK2 and cyclin E1 expressions in a dose-dependent manner ( Figure 4C,D).

### EB has no effect on cell apoptosis, necroptosis, or autophagy

To explore the mechanism of human hepatic carcinoma cell death induced by EB, we then used flow cytometry to confirm the role of EB in apoptosis. Human hepatic carcinoma cells treated with12 μM or 24 μM EB for 48 h were stained with PI or Annexin V-APC. The results showed that EB did not induce apoptosis in human hepatic carcinoma cells ( Supplementary Figure S2A,B). We also treated SMMC-7721 and HCCLM3 cells with 6 μM, 12 μM, or 24 μM EB for 48 h. Cleaved caspase-3 (C-caspase-3), caspase-3, and cleaved PARP (C-PARP) were significantly changed ( Supplementary Figure S2C), which indicates that EB does not cause apoptosis in these cells.

We also confirmed the effect of EB on necroptosis and autophagy by western blot analysis. The results showed that EB did not affect the expressions of p-MLKL, MLKL, RIP1, or the LC3-II/LC3-I ratio, which are indicators of necroptosis and autophagy ( Supplementary Figure S2D,E). To further confirm these results, we used Z-VAD-FMK (20 μM), necrostatin-1 (30 μM), or 3-MA (2 mM) in the CCK-8 assay. The results showed that Z-VAD-FMK, necrostatin-1, or 3-MA did not reverse the decreased cell viability caused by EB ( Supplementary Figure S2F). These results suggest that EB does not induce apoptosis, necroptosis, or autophagy in human hepatic carcinoma cells.

### EB induces the ferroptotic death of human hepatic carcinoma cells

**Figure 5:**
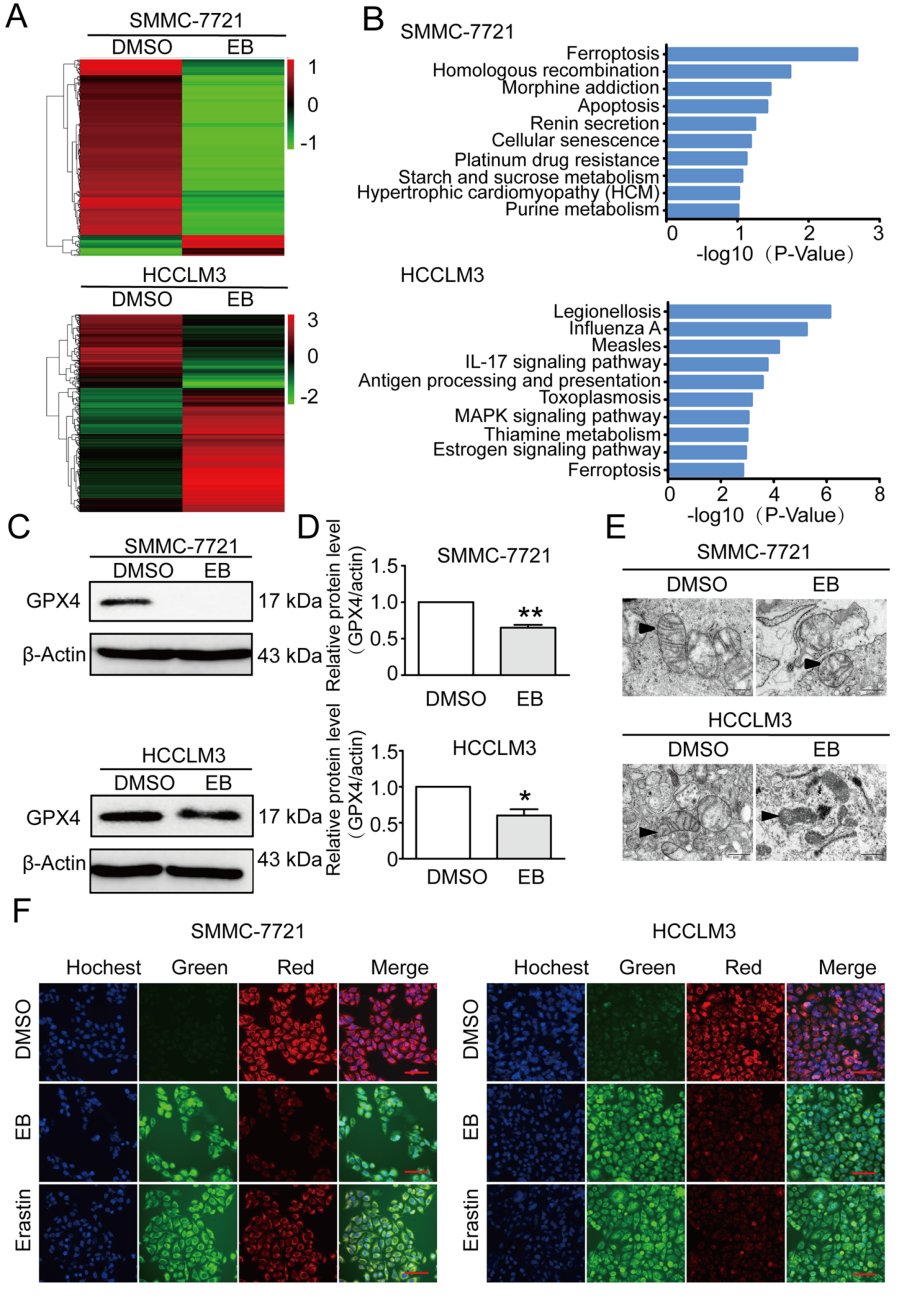
Figure 5 EB induces the ferroptotic death of human hepatic carcinoma cells (A) Heatmap showing differential gene expression in SMMC-7721 and HCCLM3 cells with or without 24 μM EB treatment for 48 h. (B) KEEG analysis of genes in SMMC-7721 and HCCLM3 cells. The top 10 or 12 biological process terms based on fold enrichment are shown. (C) Western blot analysis of glutathione peroxidase 4 (GPx4) in SMMC-7721 and HCCLM3 cells following treatment with 24 μM EB for 48 h. (D) Densitometry of western blots in panel (C). n=3. (E) Transmission electron microscopy of SMMC-7721 and HCCLM3 cells treated with DMSO or 24 μM EB for 48 h. Single black arrowheads: shrunken mitochondria. (F) EB-induced lipid ROS production in SMMC-7721 and HCCLM3 cells measured by BODIPY™ 581/591 C11. Erastin was used as positive control. All data are shown as the mean±SD. * P<0.05, ** P<0.01, *** P<0.001.

To investigate how EB inhibits cell growth, proliferation, and migration, transcriptome analysis was performed on EB-treated and control cells. Figure 5A shows differential gene expression in SMMC-7721 and HCCLM3 cells with or without EB treatment for 48 h. The KEGG results indicated that the genes upregulated by EB treatment were associated with ferroptosis under the category of cell growth and death ( Figure 5B). We then measured the expression of GPx4, a marker of ferroptosis, in cells exposed to EB, and found that EB significantly decreased the expression of GPx4 in SMMC-7721 and HCCLM3 cells ( Figure 5C,D).

The transmission electron microscopy (TEM) results showed the distinctive morphological features of EB-treated cells, in which mitochondria appeared smaller than normal, with increased membrane density, which are characteristics of ferroptosis ( Figure 5E). We further measured lipid ROS production in SMMC-7721 and HCCLM3 cells exposed to EB using BODIPY™ 581/591 C11, and found that EB increased lipid ROS levels compared with the control group ( Figure 5F). These results suggest that EB induces the ferroptotic death of human hepatic carcinoma cells.

### Heme oxygenase (HO)-1 mediates endoplasmic reticulum (ER) stress in EB-induced ferroptosis

**Figure 6:**
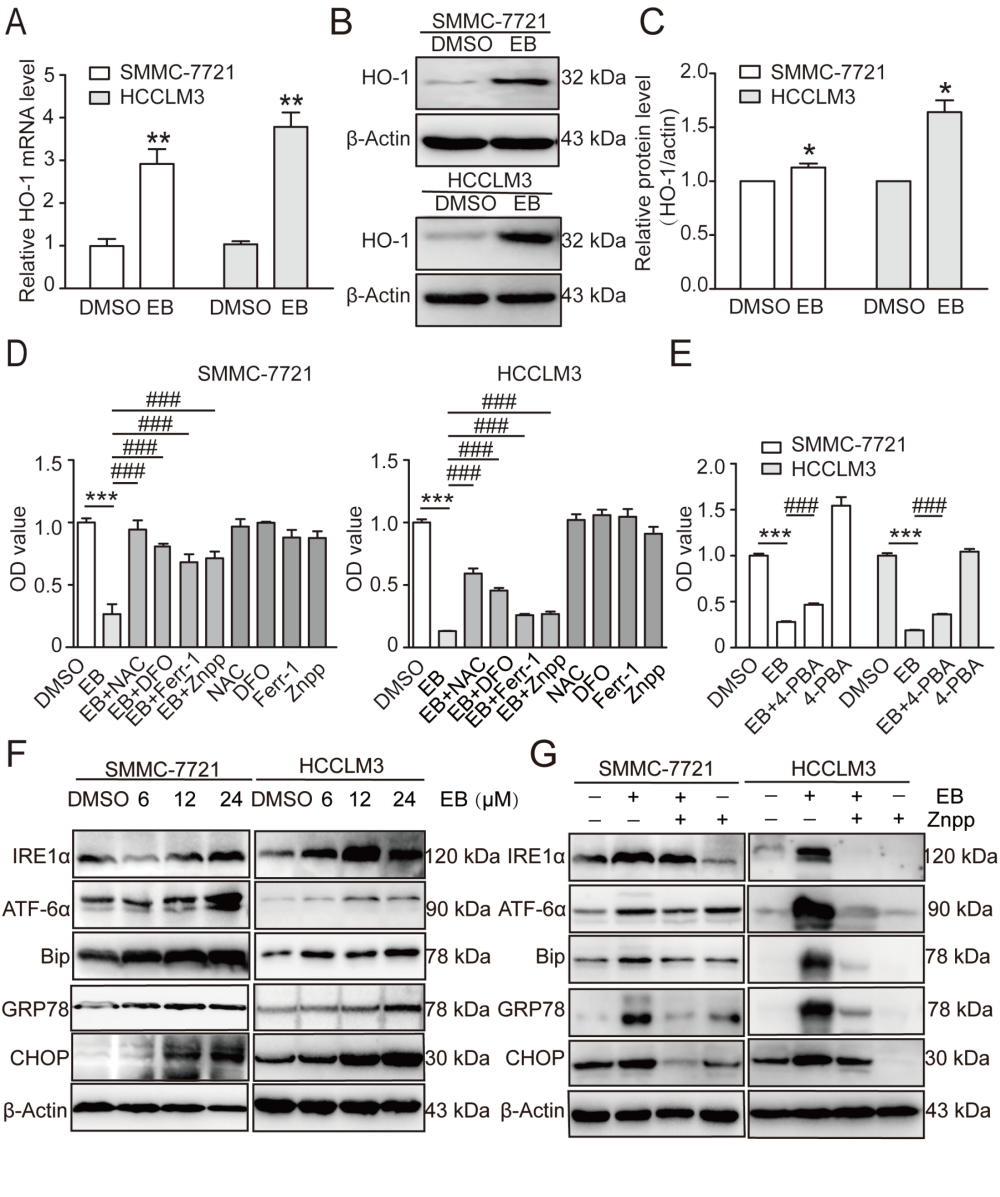
Figure 6 HO-1 mediates ER stress along with the EB-induced cell death (A) Relative mRNA expression levels measured by quantitative real-time PCR. n=3. (B) Western blot analysis of HO-1 in SMMC-7721 and HCCLM3 cells. (C) Densitometry of western blots in panel (B). n=3. (D) Cell viability assessed by CCK8 assay after incubation with ROS inhibitor NAC, ferroptosis inhibitor DFO, Ferr-1 and HO-1 inhibitor Znpp. n=6. (E) Cell viability assessed by CCK8 assay after incubation with ER stress inhibitor 4-PBA. n=6. (F) Western blot analysis of ER-related proteins GRP78, IRE1ɑ, CHOP, ATF-6ɑ and Bip in SMMC-7721 and HCCLM3 cells. (G) Western blot analysis of ER-related protein after treatment with HO-1 inhibitor Znpp. All data are shown as the mean±SD. * P<0.05, ** P<0.01, *** P<0.001; ### P<0.001 vs EB.

Transcriptome analysis indicated that HO-1, which participates in ferroptosis signaling, was significantly upregulated. To verify the bioinformatics results, we measured the expression of HO-1 by qRT-PCR and western blot analysis. The results showed that EB significantly increased HO-1 expression at the RNA and protein levels ( Figure 6A–C). When SMMC-7721 and HCCLM3 cells were pre-treated with NAC (3 mM), Ferr-1 (10 μM), DFO (100 μM), or ZnPP (10 μM) for 1 h, and then cultured with or without EB for 24 h, these inhibitors partly abrogated the decreased cell viability caused by EB ( Figure 6D). 4-PBA (1 mM) reversed EB-induced decreases in cell viability in the SMMC-7721 and HCCLM3 cell lines ( Figure 6E). HO-1 is involved in ER stress [19]. Treatment with EB significantly promoted the expressions of ER stress proteins, including GRP78, IRE1a, CHOP, ATF-6α, and Bip ( Figure 6F). Treatment with ZnPP attenuated HO-1, which resulted in ER stress protein expression induced by EB ( Figure 6G). In PDX model, EB can reduce the expression of GPX4 and increase the expression of HO-1 ( Supplementary Figure S1C). Collectively, these results demonstrated that HO-1 mediates ER stress along with the EB-induced ferroptotic process.

### EB inhibits the migration of human hepatic carcinoma cells via the ROS-ER-JNK signaling pathway

**Figure 7:**
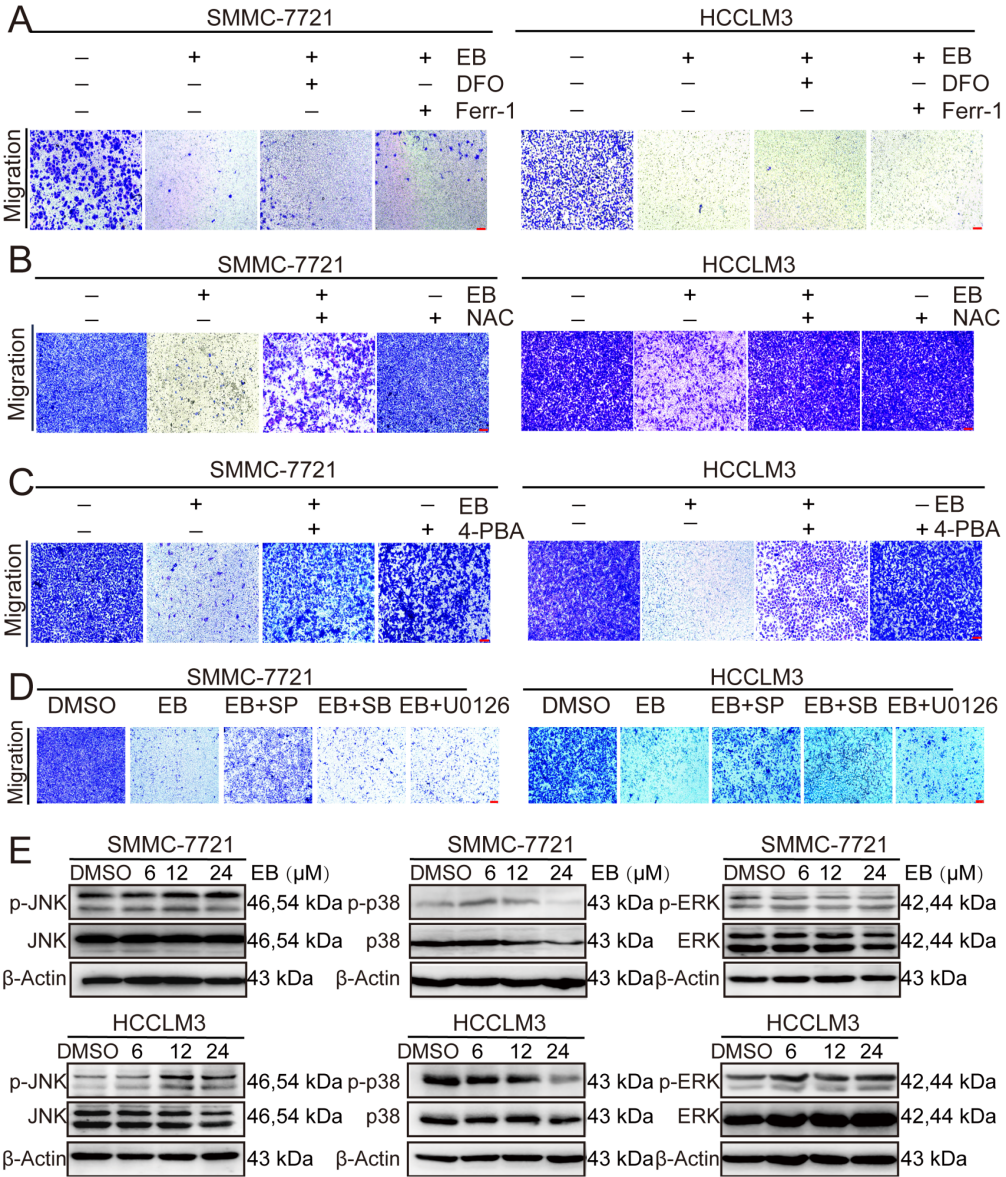
Figure 7 ROS and ER stress are involved in EB-induced cell migration inhibition (A) Effects of specific ferroptosis inhibitors DFO and Ferr-1 on EB-suppressed SMMC-7721 and HCCLM3 cell migration by transwell assay. Scale bar=100 μm. (B) Effects of specific ROS inhibitor NAC on EB-suppressed SMMC-7721 and HCCLM3 cell migration by transwell assay. Scale bar=100 μm. (C) Effects of specific ER stress inhibitor 4-PBA on EB-suppressed SMMC-7721 and HCCLM3 cell migration by transwell assay. Scale bar=100 μm. (D) Effects of specific JNK pathway inhibitor (SP600125), p38 pathway inhibitor (SB203580) and MEK 1/2 inhibitor (U0126) on EB-suppressed SMMC-7721 and HCCLM3 cell migration by transwell assay. Scale bar=100 μm. (E) Western blot analysis of MAPK (p38, JNK and ERK) proteins levels in SMMC-7721 and HCCLM3 cells.

We used ferroptosis inhibitors DFO and Ferr-1 to confirm the effect of EB on the migration of human hepatic carcinoma cells. DFO (100 μM) and Ferr-1 (10 μM) cannot reverse the migration inhibition induced by EB ( Figure 7A). However, the migration inhibition was reversed by NAC (3 mM) and 4-PBA (1 mM) ( Figure 7B,C and Supplementary Figure S3A,B). These results indicated that the mechanism of migration is related to ROS and endoplasmic reticulum (ER) stress, and is not connected to ferroptosis.

MAPKs are canonical ROS- and ER-stress-responsive signaling pathways that control cell death under oxidative stress [ 20, 21] . Therefore, the cells were treated with SP600125 (10 μM), SB203580 (10 μM), and U0126 (10 μM). Suppressing the JNK signaling pathway prominently reversed EB’s effects on cell migration at 24 h, as revealed by cell scratching assay or transwell assay ( Figure 7D and Supplementary Figure S3C).

**Figure 8:**
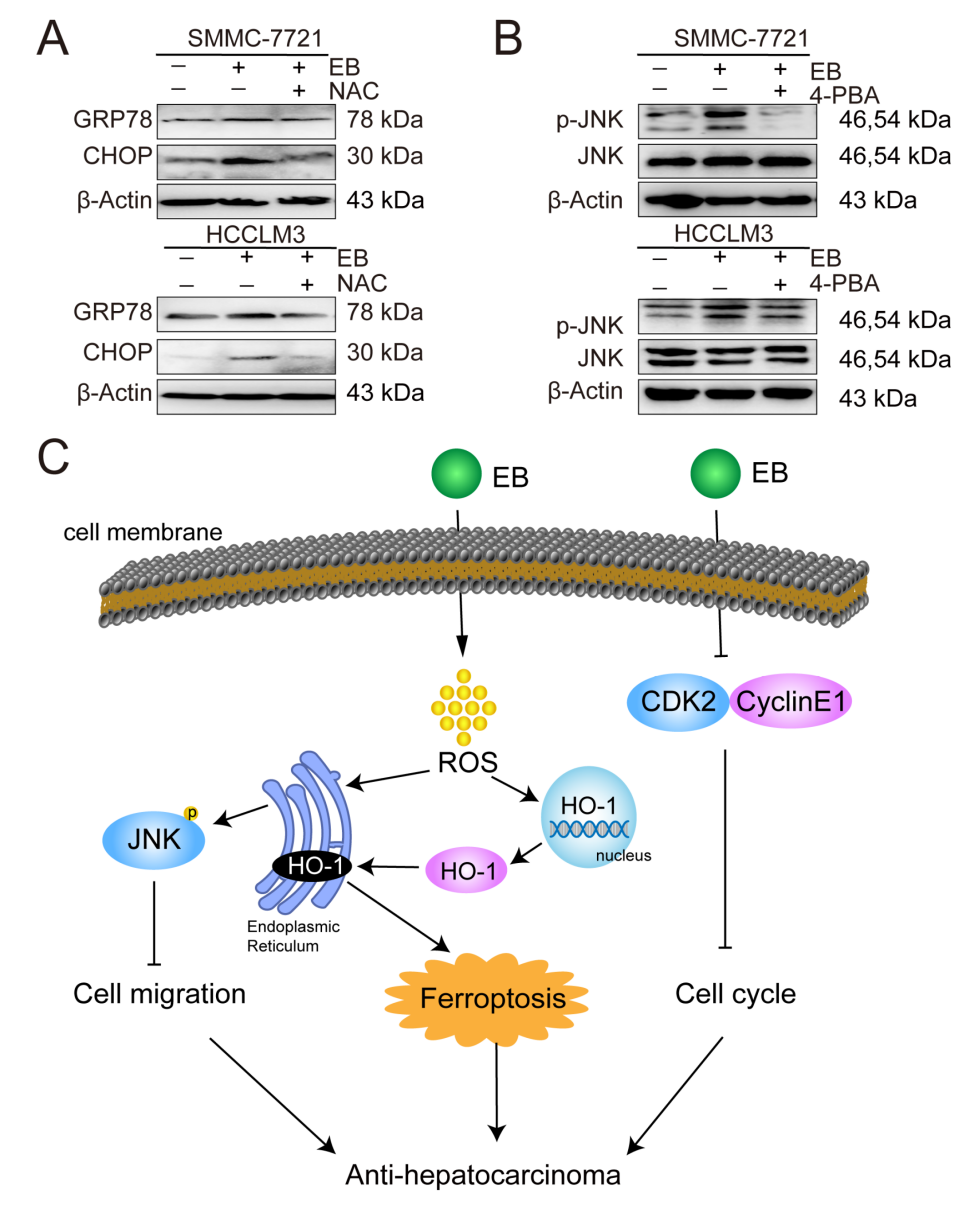
Figure 8 EB suppresses cell migration via the ROS-ER-JNK signaling pathway (A) Representative results of western blot analysis showed the effects of NAC on the expressions of specific ER stress-response proteins (GRP78 and CHOP) in EB-treated SMMC-7721 and HCCLM3 cells. (B) Representative results of western blot analysis showed the effects of 4-PBA on the activation of JNK. (C) Diagram shows that EB inhibits hepatic carcinoma by inducing ferroptosis and blocking cell cycle.

Western blot analysis revealed prominent activation of p-JNK expression in SMMC-7721 and HCCLM3 cells after EB treatment ( Figure 7E). We then verified the existence of the ROS-ER-JNK axis in EB-treated HCC cells using the corresponding inhibitors. The results of western blot analysis showed that co-incubation of antioxidant NAC (3 mM) with EB completely abolished EB-activated ER-related protein expression ( Figure 8A). 4-PBA (1 mM) produced similar inhibitory effects on JNK signaling pathways ( Figure 8B). These results demonstrated that EB inhibited the migration of human hepatic carcinoma cells via the ROS-ER-JNK signaling pathway.

## Discussion

Herein, we devoted our efforts to search for a novel target drug to treat hepatic carcinoma. We explored the role of EB in hepatic carcinoma *in vivo* and *in vitro*. We found that EB remarkably inhibited tumor growth in a xenograft model of human hepatic carcinoma cells and colony formation in *in vitro* experiments. CCK-8 assay, BrdU staining and soft agar assay showed that EB significantly inhibited cell proliferation in hepatic carcinoma, which proved the role of EB in inhibiting hepatic carcinoma.

Cell proliferation is closely related to cell cycle [ 22, 23] . In our study, EB inhibited cell proliferation by blocking the cell cycle of hepatic carcinoma cells at the S phase, and its specific mechanism involved downregulation of the expressions of CDK2 and cyclin E1.

Anti-tumor drugs can decrease the number of cells not only by affecting the cell cycle, but also by inducing cell death. Cell death can be divided into apoptosis, autophagy, and necrosis [ 24, 25] . CCK-8 assay showed that inhibitors of apoptosis, necrosis, and autophagy did not significantly reverse the decrease in cell viability caused by EB. Therefore, the inhibition of the proliferation of hepatic carcinoma cells by EB may be via non-canonical cell death, and not by inducing apoptosis, necrosis, or the autophagy signaling pathways.

Transcriptomics data indicated that the effect of EB on hepatic carcinoma cells may be through the ferroptosis signaling pathway. To verify this hypothesis, we observed mitochondrial morphological changes by TEM, ferroptosis-related protein expression by western blot analysis, ROS production by DFCH-DA assay, and cell viability reversal by ferroptosis inhibitors in EB-treated cells. The results proved that EB induces the ferroptotic death of human hepatic carcinoma cells.

Ferroptosis is a cell death mode that has recently been proposed, and its occurrence mainly depends on the accumulation of iron and reactive oxygen species (ROS) in cells [26]. Different from apoptosis, ferroptosis causes cell membrane rupture and bubbling, membrane density increase, decrease or disappearance of the mitochondrial ridge, and outer membrane breakage. In our study, the ferroptosis induced by EB could not be inhibited by apoptosis, necrosis, or autophagy inhibitors, but could be inhibited by antioxidants such as ferrostatin-1, lipostatin-1, and NADPH oxidase inhibitor, and iron chelating agents such as deferoxamine and ciclopirox amine [27].

It has been proven that many regulatory mechanisms are involved in ferroptosis, including the cystine-glutamic acid transport receptor (systemXC-), glutathione peroxidase (GPX4), nuclear transcription factor (NRF2), and heme oxygenase-1 (HO-1), as well as p53-related signaling pathways and voltage-dependent anion channels (VDACs). The transcriptome analysis report from our research group showed that EB may affect the ferroptosis signaling pathway [28]. The results of TEM showed that cell mitochondria were decreased in size, membrane density was increased, and the mitochondrial ridge was decreased after EB treatment. Western blot analysis showed that EB increased the expression of HO-1. An iron death inhibitor can reverse the drug-induced decrease in cell viability of liver tumor cells. Therefore, we demonstrated that EB acts against liver cancer by affecting the HO-1-related signaling pathway and subsequently inducing ferroptosis in tumor cells.

HO-1 is a stress response enzyme encoded by the *Hmoxl* gene, that can decompose heme into CO, Fe, and biliverdin. Biliverdin is reduced to bilirubin by biliverdin reductase [ 29, 30] . Previous studies have shown that HO-1 plays an important role in anti-oxidation, anti-inflammation, regulation of cell proliferation and death by degrading heme and its products [31]. The pathogenicity of HO-1 is closely related to iron metabolism. Chang *et al*. [32] found that BAY11-7085 (IκBα inhibitor) triggered ferroptosis through the Nrf2-SLC7A11-HO-1 signaling pathway. HO-1 plays an important role in the redox regulation of ferroptosis. HO-1 aggregates to the nucleus and mitochondria in response to BAY, promotes endoplasmic reticulum stress, causes mitochondrial dysfunction, leads to lysosome-targeted mitochondrial autophagy, and further induces ferroptosis [32]. Chung Su Wol’s research group also confirmed the role of HO-1 in ferroptosis. The expression of HO-1 is increased in ferroptosis induced by erastin, and HO-1 participates in iron-dependent lipid peroxidation, which leads to ferroptosis. Zinc protoporphyrin IX (ZnPP), an inhibitor of HO-1, was able to successfully reverse the above process [33]. In our study, we found that EB induced ferroptosis that affected hepatic carcinoma, but did not affect cell apoptosis. The ferroptosis induced by EB was mediated by HO-1 activation. EB can increase the expression of HO-1, and specific inhibitors of HO-1 can rescue EB-induced cell death. HO-1 mediates ER stress along with the EB-induced ferroptotic process. In addition to the HO-1 signal, ferroptosis induced by EB probably occurs through other signaling pathways.

The migration mechanism was proven not to be related to the ferroptotic process ( Figure 7A) because we found that EB promoted ROS accumulation and ER stress. MAPKs are canonical downstream signaling pathways of ER stress responses. By wound healing assay, transwell assay and western blot analysis, we found that the JNK signal is prominently activated and is the major ROS stress-responsive signaling pathway after EB treatment. Thus, we demonstrated that EB’s effects on cell migration are related to the ROS-ER stress-JNK signaling, Collectively, EB is able to successfully inhibit cell proliferation and migration in hepatic carcinoma ( Figure 8C).

In conclusion, we demonstrated that EB has the ability to inhibit cell proliferation and migration in hepatic carcinoma. EB is a promising anti-tumor compound for the treatment of hepatic carcinoma.

## Supplementary Data

Supplementary Data is available at *Acta Biochimica et Biphysica Sinica* online.
